# Surviving the cold: molecular analyses of insect cryoprotective dehydration in the Arctic springtail *Megaphorura arctica *(Tullberg)

**DOI:** 10.1186/1471-2164-10-328

**Published:** 2009-07-21

**Authors:** Melody S Clark, Michael AS Thorne, Jelena Purać, Gavin Burns, Guy Hillyard, Željko D Popović, Gordana Grubor-Lajšić, M Roger Worland

**Affiliations:** 1 British Antarctic Survey, Natural Environment Research Council, High Cross, Madingley Road, Cambridge, CB3 0ET, UK; 2 University of Novi-Sad, Faculty of Sciences, Trg Dositeja Obradovića 3, 21000 Novi Sad, Republic of Serbia

## Abstract

**Background:**

Insects provide tractable models for enhancing our understanding of the physiological and cellular processes that enable survival at extreme low temperatures. They possess three main strategies to survive the cold: freeze tolerance, freeze avoidance or cryoprotective dehydration, of which the latter method is exploited by our model species, the Arctic springtail *Megaphorura arctica*, formerly *Onychiurus arcticus *(Tullberg 1876). The physiological mechanisms underlying cryoprotective dehydration have been well characterised in *M. arctica *and to date this process has been described in only a few other species: the Antarctic nematode *Panagrolaimus davidi*, an enchytraied worm, the larvae of the Antarctic midge *Belgica antarctica *and the cocoons of the earthworm *Dendrobaena octaedra*. There are no in-depth molecular studies on the underlying cold survival mechanisms in any species.

**Results:**

A cDNA microarray was generated using 6,912 *M. arctica *clones printed in duplicate. Analysis of clones up-regulated during dehydration procedures (using both cold- and salt-induced dehydration) has identified a number of significant cellular processes, namely the production and mobilisation of trehalose, protection of cellular systems via small heat shock proteins and tissue/cellular remodelling during the dehydration process. Energy production, initiation of protein translation and cell division, plus potential tissue repair processes dominate genes identified during recovery. Heat map analysis identified a duplication of the trehalose-6-phosphate synthase (TPS) gene in *M. arctica *and also 53 clones co-regulated with TPS, including a number of membrane associated and cell signalling proteins. Q-PCR on selected candidate genes has also contributed to our understanding with glutathione-S-transferase identified as the major antioxdidant enzyme protecting the cells during these stressful procedures, and a number of protein kinase signalling molecules involved in recovery.

**Conclusion:**

Microarray analysis has proved to be a powerful technique for understanding the processes and genes involved in cryoprotective dehydration, beyond the few candidate genes identified in the current literature. Dehydration is associated with the mobilisation of trehalose, cell protection and tissue remodelling. Energy production, leading to protein production, and cell division characterise the recovery process. Novel membrane proteins, along with aquaporins and desaturases, have been identified as promising candidates for future functional analyses to better understand membrane remodelling during cellular dehydration.

## Background

Naturally cold tolerant organisms provide tractable models for enhancing our understanding of the physiological and cellular processes behind survival at extreme low temperatures [1]. Such information is not only of interest to ecologists, but also to the medical field of cryobiology with implications for the preservation of cells and tissues at low temperatures [2]. Within the Hexopoda, the Collembola (springtails) are particularly well studied [3,4]. They possess three main strategies to survive the cold: freeze tolerance, freeze avoidance and cryoprotective dehydration [4-6]. Whilst most cold tolerant springtails use freeze avoidance, one species, the Arctic springtail *Megaphorura arctica *(Tullberg) utilises the relatively novel strategy known as cryoprotective dehydration. In this process loss of water occurs across a diffusion gradient between the animal's super-cooled body fluids and ice in its surroundings, such that freezing point depression always exceeds the environmental temperature. Eventually the animals lose sufficient water to ensure that a freezing event cannot occur and the animals enter a state approaching anhydrobiosis [6,7]. To date, the only other animals this process has been described in is in the cocoons of the earthworm *Dendrobaena octaedra *[7], the Antarctic nematode *Panagrolaimus davidi *[8], an enchytraied worm [9] and the larvae of the Antarctic midge *Belgica antarctica *[10].

The physiological mechanisms underlying cryoprotective dehydration have been well characterised in *M. arctica *[6]. These include dehydration through a highly permeable cuticle, accumulation of trehalose as a cryoprotectant [6,11,12] and changes in membrane phospholipid fatty acid composition [13]. Trehalose accumulation has also been documented in the nematode [8]. In other species desiccation tolerance has also been correlated with these cellular processes [14-16], but additionally with redistribution of osmolytes [17] and accumulation and synergistic colligative effects of amino acids [18]. Gene expression changes have also been identified such as up-regulation of the heat shock protein genes Hsp23, Hsp70 and Hsp104 [19,20] and also genes encoding a ferritin homologue [21], the desiccation-associated LEA family [22], fibrinogen, mitochondrial transporters, acidic ribosomal phosphoprotein P0, phosphoglycerate kinase and the ribosomal protein RPL7 [[23] and references therein]. In addition, protein phosphatases, protein kinase A and p38 MAPK have been suggested as being involved in transcriptional control [24,25]. Whether these processes or genes are potentially involved in cryoprotective dehydration in *M. arctica *is, as yet, unknown, but they do provide potential targets for further investigation. However it should be noted that the examples quoted above include a wide taxonomic range (from yeast through to frogs) and also different dehydration or desiccation tolerant mechanisms, mainly diapause and rapid cold hardening, between which, even with the limited molecular investigations to date, there are documented differences in gene expression between species [26]. Currently nothing is known about the transcriptional and translational processes affecting survival of *M. arctica *at low temperatures.

In order to address this lack of data, we have generated and screened a custom-made *M. arctica *microarray. Previously we described an EST project of 16,379 clones generated from *M. arctica *in different dehydration states [27]. A sub-set of these clones (6,912) formed the basis for the production of the microarray used in this article. The aim was to identify the underlying gene expression pathways involved in cryoprotective dehydration in this organism and determine whether these pathways were exclusively up-regulated in response to cold dehydration. To this end the microarray was hybridized with RNA extracted from animals that had been treated under different regimes of both cold and salt-induced dehydration. We present the first comprehensive microarray analysis of the transcriptional responses underlying the cryoprotective dehydration process.

## Results and discussion

Animals were treated according to five protocols, the results from each of which were compared with control animals maintained at +5°C:

• -2°C animals were cold dehydrated to -2°C.

• -7°C animals were cold dehydrated to -7°C.

• H18 were -7°C animals left to recover at +5°C for 18 hours.

• 0.9 salt were dehydrated in a reduced humidity atmosphere at +5°C to a water content of 0.9 g/g dry weight.

• 0.2 salt were dehydrated as above, but to a water content of 0.2 g/g dry weight.

In all cases, survival of the animals after dehydration treatment was monitored. The

-2°C animals could still be seen to move and only living samples were selected and flash frozen. When the animals were subjected to salt and -7°C dehydration, it was still possible to see which animals were alive and had successfully undergone "protective dehydration". They were darker in colour and smaller (shrivelled). However, a subsample of these selected animals was taken back to +5°C with water and they all survived. Some animals did not successfully enter the dehydrated state and therefore died. They looked very different: long and distended and so were easy to distinguish.

**Figure 1:**
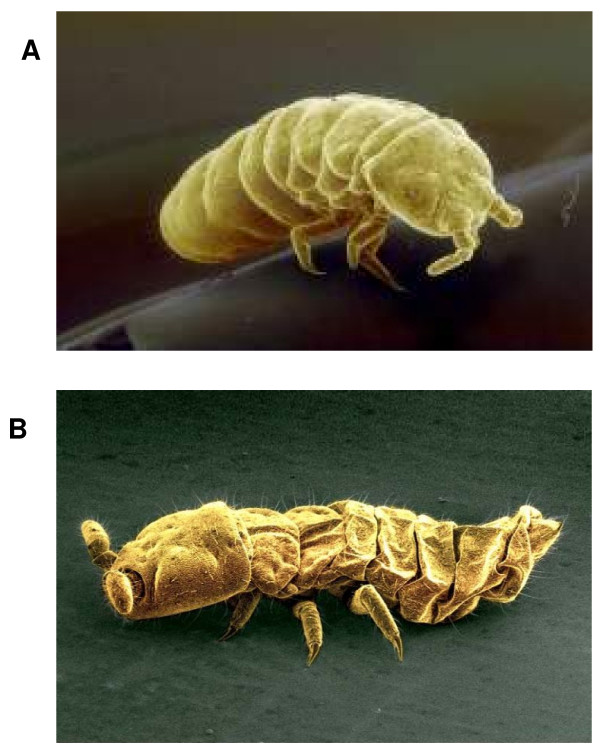
**A: SEM of *M. arctica *in "normal" fully hydrated state**. **B**: SEM of *M. arctica *in dehydrated state. Photo courtesy of K. Robinson and M. R. Worland.

**Figure 2:**
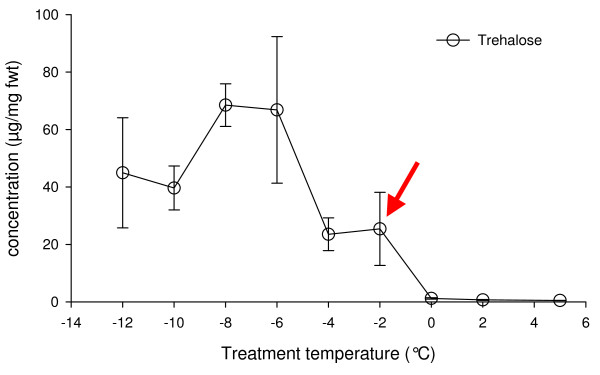
**Graph showing increase in trehalose content (μg/mg fresh weight) of *M. arctica *with decreasing temperature**. 0°C to -2°C "tipping point" is arrowed.

Analysis of the microarray results showed large-scale changes in gene expression over all treatments. This ranged from 7.4% of the genes being up-regulated with the -2°C treatment to 16.1% up-regulated with the 18 hour recovery (Table 1). When down regulated genes are also considered, the total percentage of genes changing expression levels increased to 10.8% and 32.4% respectively. In many ways this is not surprising, given that since *M. arctica *is so small, the survey of response to change has to be carried out at the whole organism level and this results in dramatic morphological and biochemical changes. The body of *M. arctica *effectively shrivels to a fraction of its former volume as the total water content of the animal reduces from 70% to 40% of fresh weight, most of which is due to the loss of osmotically active water and the animal becoming almost anhydrobiotic [12] (Figure 1). This process is accompanied by production of the disaccharide trehalose as a cryoprotectant, with temperatures of between 0°C and -2°C appearing to act as the tipping point for trehalose synthesis (Figure 2). Such changes are clearly not the result of the action of a few genes. However the problem arises as to how to analyse such a dynamic dataset to produce an overview of the transcriptional processes involved and therefore narrow down areas for future research. This is more difficult in a non-model species given that assignment of putative functionality via BLAST sequence similarity searching was only achieved in 42–57% of ESTs (Table 1).

**Table 1:** Up-regulated genes under the different treatments, detailing the percentage with putative function assigned on the basis of BLAST sequence similarity searching.

	**-2**	**-7**	**H18**	**0.9 salt**	**0.2 salt**
Up-regulated	512	914	1113	778	846

**Up-regulated expressed as % on chip**	**7.4%**	**8.8%**	**16.1%**	**11.2%**	**12.2%**

					

Unsequenced	43	84	93	57	70

Sequenced	469	857	1020	721	776

Identified	216	504	568	444	468

**% with putative ID/function**	**42.1%**	**55.1%**	**51.0%**	**57.0%**	**55.3%**

**Figure 3:**
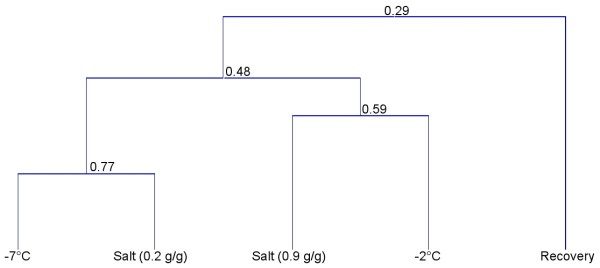
**Hierarchical clustering for 18 hour recovered animals compared to -2°C, -7°C and salt dehydrated animals**. Minimum similarity scores are shown.

To facilitate description of the processes, the analysis is presented in the two distinct components of dehydration and recovery. This is validated by hierarchical clustering which clearly shows the dehydration process as very distinct from recovery with clear partitioning of the five groups (Figure 3). Within the dehydration strategies, the 0.9 salt expression profiles cluster most closely with the -2°C group and the 0.2 salt with the -7°C group, which would be expected on the basis of water content, if the molecular processes involved are similar in both cold and salt dehydration.

### Gene ontology overview

**Figure 4:**
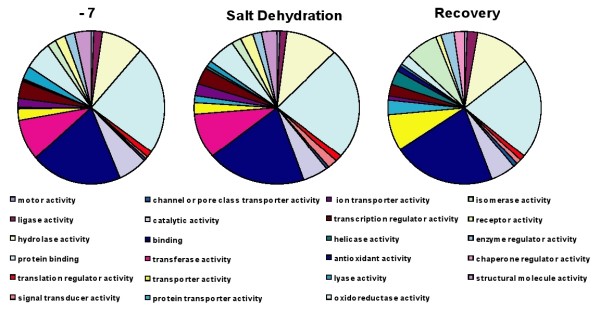
**GO Slim analyses of gene expression in 18 hour recovered animals, -7°C cold and 0.2 salt dehydrated animals**.

To establish an initial overview of the processes involved and to potentially differentiate between cold and salt dehydration stresses, GO and GO Slim ontology enrichment analyses were used. When GO Slim (Molecular Function) was used to compare gene ontology in control animals compared with those treated at -7°C and also those of the 0.2 salt group, there was virtually no difference in the relative partitioning between different functions (Figure 4) under the two different treatments. So although the number of up-regulated clones varied between the control animals and both cold and salt dehydration (914 and 846 respectively), the overall functions involved in the dehydration processes appear to be very similar under the two different treatments. The reason for some disparity in the expression profiles of some genes between these two types of treatment could be due to the relative rate at which each type of dehydration reaction was achieved. The salt dehydration was experimentally induced at a faster rate, for example approximately 8 days to dehydrate to 0.9 g/g dry weight with salt compared to 24 days to reach 1.1 g/g dry weight with the cold treatment. So the slight discrepancy in the dry weight levels, the production methods used, different stresses and the fact that the experiments proceeded at different rates, could explain differences in the fine-scale detail. Therefore although the relative fine-tuning of gene expression could be affected, the same final result would be achieved in both cases. Indeed previous observations suggest that desiccation and cryoprotective dehydration mechanisms are very similar [28] and that there is overlap in tolerance pathways to different stress factors [29-31].

The GO Slim analysis of the 18 hour recovery group was also very similar to the dehydrated states (Figure 4). GO enrichment analysis comparing gene ontology in control animals with the -7°C and 18 hour recovery groups (data not shown) revealed no real enrichment for particular GO categories. GO analysis can produce a very broad overview of functions and processes which change in organisms under different experimental conditions. Whilst this is useful and indeed can indicate potential clones to study in more detail (c.f. [27]), to more accurately define what is happening at the molecular level, actual gene identifications are required. Because the expression profiles of so many genes change under the different experimental scenarios used here, it was decided to analyse, in detail, the "Top 20" most highly expressed clones under each of the different conditions (compared to the control group).

### Dehydration processes using putative gene identifications

Of the 512 clones up-regulated during the -2°C treatment, only 42.1% could have some putative functionality assigned via BLAST sequence similarity searching (Table 1). This is a similar percentage when the top 20 from this listing are examined [Additional file 1]. Not surprisingly TPS is significantly up-regulated and of particular interest is the match to the *Drosophila *Mediator complex. This is one of the central players in a class of regulatory coactivator complexes that potentiate activated transcription and fine tune the transcriptional response so that it is physiologically appropriate [32]. Given that such a dramatic reduction occurs in water content, it is not surprising to find the presence of a small heat shock protein with homology to the Lethal (2) essential for life protein (HSP20 family) from the yellow fever mosquito, *Aedes aegypti*.

A number of clones have been designated as "bacterial contamination" in Additional files 1, 2, 3, 4. These sequences all belonged to a single 2 kb contig of 14 clones matching bacterial isocitrate lyase (accession number: A9IUW1). There is some debate as to the presence of isocitrate lyase in the Metazoa, with evidence from database analyses of identification only in nematodes and the cnidarians [33]. In this analysis [33], putative isocitrate lyase genes were described from the insects *Aedes aegypti *and *Anopheles gambiae*. These were designated as bacterial contamination due to an absence of introns and strong conservation of amino acid sequence between the putative Metazoan genes and *E. coli*. The contig from the *M. arctica *EST collection shows high sequence identity to bacterial sequences (71% identity and 83% similarity at the amino acid level) and PCR of an 800 bp fragment from both genomic DNA and cDNA revealed no difference in the size of products i.e. no indication of the presence of introns. Hence it was decided that this evidence strongly indicates that these clones were of bacterial origin, a potential hazard when producing ESTs and screening whole animals.

Examination of the ESTs most up-regulated at -7°C [Additional file 2] shows a transition from energy and trehalose production to trehalose mobilisation and cellular/skeletal reconstruction. Of prime interest is the match to the *Anopheles gambiae *trehalose transporter (AgTRET1, accession number: A9ZSY1), which according to the database entry, is responsible for the discharge of trehalose from the fat body into the haemolymph (Kanamori *et al*, unpublished). This has been confirmed by functional analysis of TRET1 in the anhydrobiotic insect, *Polypedilum vanderplanki *[34]. The other matches to inosine 5' monophosphate dehydrogenase (IMPDH), AKT2 (Rac serine/threonine protein kinase), F-capping protein, ubiquitin carrier protein (UCP) and transcriptional endoplasmic reticulum ATPase (TER94) are all either involved in cell growth and development, cytoskeleton or protein quality control. Overall this presents a picture of cellular/tissue remodelling, which involves production, but also degradation of protein elements. Certainly knock-out/mutation experiments in both the capping protein in *Drosophila *and AKT in mice has resulted in severely premature death with skeletal and developmental defects [35,36]. Potential controlling elements in these processes are indicated by the continued presence of matches to the Mediator complex and also juvenile hormone esterase, an enzyme regulating the levels of juvenile hormone, a key hormone in insect development and reproduction [37].

Given experimental differences and inter-experimental variation, it would not be expected that the clones up-regulated in the salt dehydration experiments exactly mirror those induced by cold. The gene identifications are more diverse with the 0.9 salt data compared to the other experiments, but do not alter radically from the trends seen previously [Additional file 3]. There is still up-regulation of TPS and modification of transcription levels via the Mediator complex [Additional file 3]. Identifications indicate that the processes of cellular reconstruction and proteolysis are still on-going, but additional signalling molecules are apparent in the list. The latter include matches to serine/threonine protein kinases (unc-51 from *C. elegans *and itk, a Tec-family kinase from *Danio rerio*). Modification of the protein pool and cellular remodelling is still taking place with the presence of a co-chaperonin (TCP-1), a proteasome 26s ATPase sub-unit, matches to an autophagy protein from the fathead minnow and a long, if weak but consistent match to a large number of uncharacterised proteins which include the tolloid protein from the pacific oyster (*Crassostrea gigas*) with a protease function and a nucleolysin TIAR protein, which is a cytotoxic granule associated RNA binding protein involved in targeting apoptosis.

Despite a relatively large number of clones with no sequence data in the 0.2 salt table, the same gene identifications emerge with significant up-regulation of TPS, the Mediator complex and the protein kinase AKT2 [Additional file 4]. One clone, sb_009_04J09 was labelled as a putative transporter. This clone produced weak matches (39% amino acid identity) to a number of different clones including the *Culex quinquifasciatus *chromaffin granule amine transporter (BOXJX9), an uncharacterised MFS-type transporter (Q6NT16) in human and a novel facilitator super family protein in *Xenopus tropicalis *(Q07G00). So although an orthologous gene was not identified, this clone potentially represents an interesting candidate for further analysis with a putative transporter function.

### Recovery process definition using putative gene identifications

The profile of the ESTs associated with this treatment [Additional file 5] is very different to those of dehydration with an emphasis on energy generation and transcription processes, but again there is an element of cytoskeletal reconstruction. Of primary note is an EST with sequence similarity to arginine kinase. Arginine kinase is a phosphagen kinase, these enzymes are prevalent in systems with fluctuating energy demands, acting as an energy buffering system [38] and acts as an energy shuttle delivering ATP generated by mitochondria to high energy requiring processes, such as membrane turnover [39], crucial when emerging from a dormant state. Whilst the phosphagen kinases are a multigene family, arginine kinase is the only form of this gene in arthropods and molluscs [40]. It also has other functions such as buffering intracellular pH and preventing a rise in intracellular ADP levels that would trigger multiple metabolic responses. Clearly there will also be a need for *M. arctica *to redevelop the internal cellular structures, repairing any that have been damaged in the dehydration process [41,42], hence the presence of putative chitin binding proteins and proteases. Alongside this are matches to genes associated with the cytoskeleton via microtubules and mRNA translation. These are represented by a member of the tektin protein family (microtubule associated cytoskeletal proteins), a deep, but weak match to the eukaryotic initiation factor 4γ and an elongation factor 1 α-like factor. The latter is a polypeptide chain release factor active during mRNA translation, but is also involved in the microtubule cytoskeleton and has been implicated as playing a role in cell division [43].

Other putative identifications have less obvious functions when considered in the light of springtail physiology, including a match to leukotriene A-4 hydrolase. This is a dual purpose protein with both amino peptidase and epoxide hydrolase activity. This gene has been well characterised in human and is involved in inflammation and host defenses [44], although given the differences between humans and springtails, its exact role in *M. arctica *will need further analysis. There are also a couple of weak matches (approximately 7.0 e^-09^) to a high mobility group protein B1. This gene is involved in the regulation of transcription and interestingly was implicated as an important element in the response of *Austrofundulus limnaes *(annual killifish) to fluctuating daily temperatures [45]. The *M. arctica *gene is not an orthologue of this sequence, but such a match could imply a similarity of function.

As for potential hormonal control of the rehydration process, juvenile hormone is not surprisingly implicated (given its pleiotropic activities). There is a match to accession number: Q5XUU6, which is described as a Take-out-like carrier protein (JHBP-1) with a juvenile hormone binding motif (Kucharski and Maleszka, unpub).

**Figure 5:**
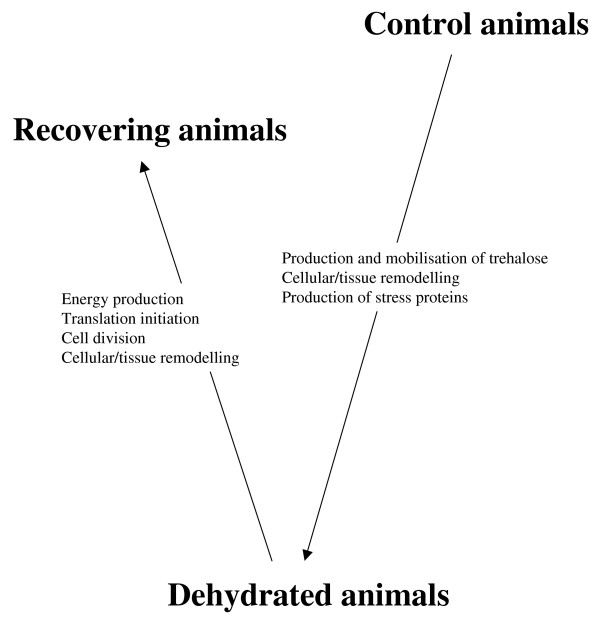
**Overview of processes involved in *M. arctica *dehydrated and recovering animals**.

Although this analysis was restricted to the "Top 20" up-regulated clones under each treatment, it is possible to identify major changes in springtail cellular processes associated with these experimental conditions (Figure 5). However, these represent restricted snapshots of gene expression of processes, which range from hours (recovery from cold dehydration) to several weeks (reduction of temperatures to remove all osmotically active water). They also do not represent a comprehensive time course series, which could potentially add detail to the more global observations made here. When considering genes that may be co-regulated with anhydrobiosis, there is an obvious process, which is known to correlate with dehydration in the Arctic springtail; that of trehalose accumulation.

### Genes associated with trehalose metabolism

**Figure 6:**
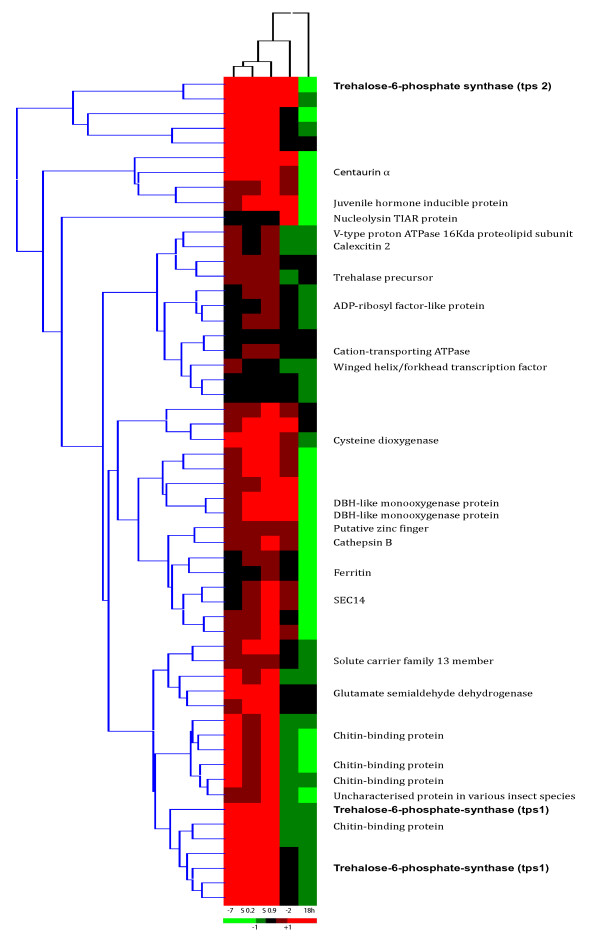
**Heat map produced via identification of genes co-regulated with TPS in all 4 dehydration treatments**.

The process of dehydration is accompanied by a concomitant increase in the levels of the carbohydrate trehalose, which is presumed to also act as a cryoprotectant in this organism. The levels of trehalose increase dramatically in the animal when the temperature reaches -2°C and continues as the temperature drops, reaching a plateau at -6°C (Figure 2). During this reduction in temperature, the trehalose concentration increases 100 fold from 0.9 to 94.7 μg/ml and is clearly a critical pathway in the dehydration process [12]. Therefore, in order to target more accurately genes specifically involved in dehydration, clones, which co-expressed with trehalose-6-phosphate synthase (TPS), were identified in the microarray data analysis (Figure 6). This was complicated slightly by the identification of a gene duplication event for TPS.

### Duplication of TPS genes in M. arctica

Examination of clone matches to TPS revealed 2 separate contigs, representing distinct paralogues:

• Contig 1 comprised 2 clones (sb_006_04P17 and sb_009_07N11) with a total length of 748 bp. BLAST sequence similarity searching produced database matches with a score of 983, a probability of 5.7e^-97 ^and an 86% match to accession number A8D372 from *Locusta migratoria malinensis *(Oriental migratory locust). Both of these clones were present on the microarray.

• Contig 2 comprised 4 clones (sb_008_04E04, sb_006_06G01, sb_007_11P11 and sb_005_09H16 with a total length of 631 bp. BLAST sequence similarity searching produced closest database matches to A3EY17, a fragment of TPS from *Maconellicoccus hirsutus *(pink hibiscus mealy bug). The first match to a full-length sequence was to *Drosophila sechellia *(B4I383) with a score of 413, a probability of 9.7e^-36 ^and 66% identity. Only one of these clones (sb_006_06G01) was present of the microarray).

Both contigs overlapped the same region by the order of 100 amino acids, sharing 42.1% identity and 57.9% similarity at the protein level. The duplication of TPS genes in *M. arctica *mirrors the situation found in the nematode *C. elegans *[46] and the rotifer *Brachionus plicatilis *[47]. Direct sequence comparisons of the duplicated genes are difficult between these organisms. The *C. elegans *sequences are much longer at 1229 and 1331 amino acids, compared to the insect sequences (at approximately 800 amino acids), which provide the closest database matches to the *M. arctica *sequences. Both putative rotifer genes are fragmented with only one encompassing the same coding region as the two *M. arctica *sequences. This fragment shows between 33–36% sequence identity at the amino acid level with the two springtail sequences. This low level of homology is not surprising as these sequence fragments comprise the most 3' end of the gene, a region which is invariably less conserved. This would also explain what appears to be the relatively low level of homology between paralogous springtail genes (42.1% identity), compared with those of the rotifer which share 88.9% identity, as the rotifer sequences are compared from the middle, more conserved portion of the gene. The *C. elegans *clones shared only 48% identity over their whole length.

As regards the putative functionality of the duplicated TPS genes; in *C. elegans *trehalose is also accumulated in the animal in response to stressful conditions, such as heat shock and freezing. However, RNAi knockout experiments of the two TPS genes produced no obvious short-term loss of function in this organism, despite in vivo levels being reduced to 7% of normal in the double knock-out [46]. The rotifer (*B. plicatilis*) regularly survives stressful conditions via the production of resting eggs which undergo obligatory diapause or dormancy, but trehalose does not appear to accumulate in this animal under such conditions [47]. However small amounts of trehalose (0.35% dry weight) have been found in *B. plicatilis *anhydrobiotic resting eggs [48], but may be acting as an osmotic electrolyte rather than a cryoprotective chemical [48]. The fact that duplicate genes for TPS exist implies a partitioning of function between the two paralogues [49], indeed as will be seen below, there is differential expression of the two genes in *M. arctica *under dehyrating conditions.

Similarly to the duplication of the TPS gene in *M. arctica*, there appears to be (again, in line with the situation in *C. elegans *and the rotifer, *B. plicatilis*) a parallel duplication of the trehalase gene. To date there are 4 trehalase genes annotated in the whole genome assembly of C. elegans (W05E10.4, F57B10.7, T05A12.2 and C23H3.7) and 3 putative gene fragments were identified from the rotifer EST project [41]. Although the EST coverage of *M. arctica *is lower than the rotifer, 3 singletons were identified as putative trehalase genes. These three sequences had sufficient differences (between 54.4% and 68.3% identity at the amino acid level) to define them as putative paralogues. To date RNAi studies in *C. elegans *have produced no obvious phenotype, so the question remains as to why there are four copies of this gene in *C. elegans *and also multiple copies in other organisms.

### ESTs co-regulated with TPS

In total 53 ESTs were significantly co-regulated with the 3 TPS sequences (Figure 6, Additional file 6). Of the 56 clones, it was possible to ascribe putative function via BLAST homology to 26 clones (including the 3 TPS ESTs) (46% of total), no significant database match was identified for 25 clones and 5 had no sequence data attached. In each case, assignment of putative functionality was accorded when probability values were in excess of 1 e^-10^, unless specifically stated in the discussion.

Putative functions of a major sub-group of ESTs centre around cellular signalling and membrane trafficking. The ADP-ribosylation-factor family (ARF) are Ras-related small GTPases that regulate intracellular trafficking by shuttling between an inactive GDP-bound and an active GTP-bound form. Two members of this family were identified in the ESTs (ADP ribosyl factor-like protein and centaurin α) [50,51]. Additionally SEC14 is a cytosolic protein with the ability to catalyse exchange of phospholipids between membranes, acting as an essential interface between lipid metabolism and membrane trafficking in the trans-golgi complex [52]. Interestingly, this gene is also up-regulated in yeast stress experiments involving heavy metals, alkylation and temperature (c.f. [53]). Two ESTs both show similarity to Dopamine β-monooxygenase (DBM), an enzyme, which produces norepinephrine, which is stored in secretory granules and used for intracellular communication [54]. Certainly in mammals norepinephrine is well known as both a stress hormone and neurotransmitter, although an equivalent hormone in insects is as yet uncharacterised and therefore the function of a DBM homologue is unknown in insects.

Four further ESTs are putatively involved in membrane transport: sb_006_04L08 belonging to the cation transport ATPase family, sb_006_03D06 matching caltexin, which is a calcium activated signalling molecule, which maintains a buffered intracellular Ca^2+ ^concentration and is involved in the regulation of K^+ ^channels [55], sb_006_03F06, a vacuolar ATPase involved in ion regulation and maintenance of homeostasis [56] and finally sb_009_05K08. This latter clone putatively encodes a member of the solute carrier family 13, member 5 Na^+^/citrate co-transporter. This family encode integral membrane proteins that mediate the uptake of a wide variety of molecules, with concomitant uptake of Na^+ ^ions. They may act in tandem with aquaporins, which given their function are clear target molecules in the dehydration process. This family 13 of transmembrane proteins have important physiological functions and may facilitate the utilisation of citrate for the generation of metabolic energy via glycolysis and gluconeogenesis [57,58].

Given that TPS is involved in dehydration, it is not surprising there are BLAST matches to proteins with oxidoreductase activity, which may be involved in the stress reaction, such as ferritin and cysteine dioxygenase. A ferritin-derived homologue, artemin has been identified as a major component in the encystment and diapause of the brine shrimp, *Artemia franciscana *with proposed chaperone activity and a stress protection role [21]. However artemin shows very limited identity to ferritin (20.4% identity to Q6WNX4, *Boophilus microplus *(cattle tick)) and the EST matches for *M. arctica *are clearly more similar (74% identity) to the parent ferritin gene. Cysteine dioxygenase is involved in cysteine catabolism, the relevance of which, in this situation, is unclear, but this gene has been shown to be up-regulated in response to stress (hypoxia) in the goby fish, *Gillichthys mirabilis *[59].

There are also proteins which have putative functions involving skeletal reconstruction. There are four matches to a chitin-binding protein in *Lutzomyia longipalpis *(sand fly) (accession number A8CWD0). This protein has been identified as a peritrophin and has been shown to be secreted in the mid-gut in response to feeding, but also has a cytoskeletal role [60]. These may be needed to repair cell structure as a result of dehydration. Of the remaining identifications, sb_006_08J23 shows similarity to glutamate semialdehyde dehydrogenase, which is involved in amino acid biosynthesis, and sb_006_01B14 encodes a trehalase homologue. The presence of both TPS and trehalase in the same expression profile is not surprising as there is presumably some measure of dynamism between synthesis and breakdown of trehalose. Cathepsin B is involved in proteolysis. RNAi experiments of this gene in the silk worm, *Bombyx mori *showed a significant role in tissue restructuring, being critical for metamorphosis and normal development [61]. Allied to this is the identification of a putative Nucleolysin TIAR protein, which is a cytotoxic granule associated RNA binding protein involved in targeting apoptosis. The significant presence of both genes indicates that the pair may act in tandem during skeletal restructuring. All of these genes need to be regulated and clues to the mechanism may be found in the identification of an EST with sequence similarity to a juvenile hormone inducible protein (of unknown function) (accession number: B0WJH4). Juvenile hormone is known as a pleiotropic master hormone and along with 20-hydroxyecdysone (20E) governs most aspects of insect interaction with the ecosystem, affecting decisive life history parameters such as growth, development and reproduction [62,63]. Finally, there are two clones: clone sb_009_06O13 which shows sequence similarity (3.5 e-19) to the seven-in-absentia homologue (SIAH) from *Drosophila melanogaster *and a weak, but consistent match to the winged helix/forkhead transcription factor. The SIAH gene is highly conserved between *Drosophila *and mouse [64], but from the *M. arctica *and *Drosophila *alignment, this is clearly not the case and therefore the EST cannot be designated a putative SIAH orthologue. However, SIAH does contain a zinc finger domain and therefore this EST has been putatively designated as a zinc finger protein, the members of which interact with many transcription factors and regulatory proteins. As regards the weak match to the winged helix/forkhead transcription factor, whether this is an orthologue is difficult to determine, as this gene is not highly conserved between species (approximately 30%, data not shown), but the match may indicate a putative transcription factor along similar lines to SIAH discussed previously.

This type of analysis clearly identifies a number of candidate sequences of both putatively assigned and unknown function for future investigations in the Arctic springtail. Although the identified clones co-regulate with TPS, they may not be directly relevant to TPS metabolism and so further analyses of these sequences may have wider applications to the study of dehydration processes, even in those organisms that undergo anhydrobiosis in the absence of trehalose accumulation.

### Trehalose and dehydration strategies

A significant part of this paper has been concerned with trehalose metabolism and associated gene expression analyses. Indeed, there has been a significant amount of research into the role of disaccharides as cryoprotectants and trehalose in particular [65]. Accumulation of this molecule has been shown to correlate with anhydrobiosis in a number of species (other than *M. arctica*) such as cysts of the brine shrimp *Artemia franciscana *[66], the larvae of the chironomid midge *Polypedilum vanderplanki *[67], the tardigrade *Adorybiotus coronifer *[68] and several nematode species [69-72]. However, there are also a number of examples where species undergo anhydrobiosis in the absence of disaccharide accumulation including bdelloid rotifers [48,73] and some tardigrades [74]. Also the model species of bakers yeast *Saccharomyces cerevisiae *and the nematode *C. elegans *accumulate trehalose in response to stressful conditions, but knockout experiments using TPS genes has shown very little, if any effect [46,75]. This conflicting data suggests that trehalose may not be the single most important molecule in determining whether an animal survives dehydration, but is one of a series of mechanisms, several of which may act synergistically to improve survival [74]. This emphasises the need to conduct large-scale gene expression experiments on these organisms to uncover the complexity of such interactions. In those organisms where sequence data is limiting, relatively simple statistical correlations, such as the heat map results presented here can provide valuable clues for further analyses. Where draft genome data or large-scale transcriptomic datasets exist, then more complex cellular gene network analyses are possible [76].

### Candidate gene approach using Q-PCR analyses

Having just described the advantages of developing a network approach to expression analyses, in non-model species, there is still added value in the candidate gene approach. This is particularly true where candidate genes have been investigated in other species and have been shown to have significant effect on the process under investigation (c.f. small heat shock proteins and desiccation tolerance [77]). Because the Arctic springtail experiments do not incorporate a time course experiment, it is perhaps not surprising that none of the obvious candidates such as the heat shock protein, HSP70 and the aquaporins, are featured in the bioinformatics analyses detailed above. As part of the microarray validation 21 dehydration candidate genes (based on literature searches) were chosen for more detailed Q-PCR analysis.

### Q-PCR of candidate stress genes

Q-PCR was performed on all treatments (Table 2). The first candidates were aquaporins, transmembrane proteins involved in solute transport [78]. Three aquaporin genes have been identified in *M. arctica *[27] and these were generally down regulated, except for AQPA during recovery and 0.2 salt and AQPC during the preliminary stages of cold dehydration. It is not known which solutes these springtail aquaporins transport and so they may not be the main cellular transporters or may act in concert with other transmembrane proteins. Data from *Polypedilum vanderplanki *indicates that of the two aquaporins isolated from this organism, one is involved in anhydrobiosis, whilst the other controls water homeostasis of the fat body during normal conditions [79]. So the three springtail genes could be activated under very different conditions or developmental stages. The TPS heat map results (detailed above) did identify a member of the solute carrier family 13, which has been proposed to act in tandem with aquaporins. Studies using immunoblotting with human AQP antibodies in the goldenrod gall fly (*Eurosta solidaginis*) [80] showed a similar complex picture, with upregulation of AQP3 and down regulation of AQP2 and AQP4 associated with dehydration. However, because of the different methods used (Q-PCR and immunoblotting) it is not possible to correlate these patterns of AQP expression between the two insects.

**Table 2:** Q-PCR results showing expression changes in 21 genes under cold and salt dehydration conditions plus 18 hours recovery compared to a control sequence.

	**-2**	**-7**	**0.9 salt**	**0.2 salt**	**18 hr**
**Aquaporins**					

AQP-A (clone ID: sb_006_02P03)	-1.50	-3.99	-3.27	+1.89	+1.74

AQP-B (clone ID: sb_006_05H07)	-1.67	-2.50	-1.54	-1.67	-1.89

AQP-C (clone ID: sb_006_08O08)	+1.05	-1.25	-2.00	-1.12	-1.04

					

**Heat shock proteins**					

sHSP	+4.65	+5.92	-4.18	-1.35	+8.00

HSP70	+1.06	-2.08	-2.00	+2.00	+1.14

					

**Antioxidants**					

Superoxide dismutase	-1.62	-2.31	-1.52	-1.50	-1.84

Catalase	+3.47	-1.50	-4.55	-2.60	-1.19

Glutathione-S-transferase	+37.64	+7.66	-1.50	+3.34	+21.27

					

**Various**					

Ferritin	+1.87	+2.57	-1.40	+1.19	+1.47

Desaturase	+3.42	+5.07	+3.45	-1.50	+6.32

					

**Trehalose GO annotations**					

Trehalose 6 phosphate synthase (1)	+3.57	+3.50	+1.35	+1.94	+3.68

Trehalose 6 phosphate synthase (2)	+6.08	+2.25	+10.10	+15.34	+10.60

					

LATS tumour supressor	-1.15	-2.11	-2.04	-1.30	-1.07

Serine/threonine protein kinase 38	+1.04	-1.17	-1.74	-1.33	+1.61

Protein kinase A cAMP dependant catalytic sub-unit	-1.15	-2.15	-2.05	-1.33	-1.07

Trehalase precursor	+2.26	+2.28	-2.24	-1.47	+3.35

P70 ribosomal protein S6 kinase	-1.14	-1.30	+1.26	-1.10	+2.47

Putative protein kinase DC2	+1.18	-1.08	-1.30	-1.13	+1.35

Similar to serine/threonine protein kinase 6 (Aurora family kinase 1)	-3.59	-1.89	-2.68	+1.13	+2.75

Protein kinase C	+1.08	-1.51	-2.58	-1.07	+1.43

cAMP dependant protein kinase C1	-3.19	-4.23	-2.01	+1.18	+1.46

The actual change in gene expression is given as calculated via REST [106].

The next set of candidates, the heat shock proteins showed up-regulation in cold and recovery samples, but not salt dehydration for a small heat shock protein and very little change in the level of HSP70. The up-regulation of the small HSP is also shown in the genes most highly up-regulated in the -2°C treatment. Although HSP70 is classically up-regulated in response to stress [81], this is only one of a whole family of stress-associated proteins and it may be that the expression levels of this particular member is relatively unaffected under these circumstances. Indeed multiple members of the inducible HSP70 family with differing expression patterns have been previously identified in Antarctic invertebrates [82].

Antioxidants are one of the main candidate groups proposed to be active during stressful physiological events. Of the three genes tested, glutathione-s-transferase is clearly the major gene involved in both dehydration and recovery, whilst catalase showed some up-regulation under initial cold dehydration. Allied to this, with oxidoreductase activity, was the almost universal up-regulation of ferritin (which was also identified in the heat map analysis using TPS). Not surprisingly desaturase, which is involved in membrane composition, was up-regulated in most conditions.

Finally a series of candidate genes were chosen based on their GO (Molecular Function and Biological Process) association with trehalose metabolism [27]. This list mainly comprised TPS, trehalase and a number of putative signalling molecules (protein kinases). Both TPS and trehalase genes show up-regulation under all conditions. Trehalose is important as a cryoprotectant and such a pattern of gene expression is presumably indicative of an equilibrium reaction state, with trehalose being continually produced and broken down. What expression levels do not show is how these translate into the protein production of these enzymes, the relative stabilities of the different messages or indeed the cellular requirements for trehalose in terms of timescale related to the dehydration and rehydration processes. As could be predicted, the two TPS genes show different levels of expression due to their retention via sub-functionalisation [49]. As regards the protein kinases, these tend to be up-regulated with recovery, but this may simply be a factor of timescales, with recovery happening over 18 hours, when signalling events would be more apparent than after the days and weeks it took to produce the different dehydration states. However, the fact that most of these genes did change their expression levels under at least one of the treatments, validates the use of Gene Ontology for identifying candidate genes under broad headings such as trehalose metabolism, rather than only studying very specific segments of a biochemical pathway, which require previous knowledge of the system. There are some differences between the absolute gene expression levels of the cold and salt dehydrated animals. However, as stated earlier, this could be due to different experimental conditions and variances of biological replication. The differences are not so large as to be able to differentiate between the stresses involved in cold and salt dehydration, but such minor differences point to candidate genes for further analyses during a time course experiment.

Two further obvious candidates for Q-PCR analyses are LEA proteins (c.f. [22]) and, because cold is involved, antifreeze proteins. Although antifreeze proteins have been identified in other insects [83], some of which have been shown to be very potent [84], a comprehensive analysis of our EST dataset revealed no significant matches to known antifreeze protein genes (data not shown). Hence it is presumed that antifreeze proteins do not play a role in cryportoective dehydration in the *M. arctica*. As regards LEA proteins, a group 3 LEA protein has been fully characterised in *M. arctica *and correlated with the drought response [85]. This data was published just after our paper had been submitted. Of the *M. arctica *EST clones identified as being part of the group 3 LEA gene, only one (sb_009_02E03) was present on our microarray. Unfortunately the hybridization results of this clone were not entered into the global microarray analysis because of quality control issues and therefore it was not possible to correlate the *M. arctica *microarray expression results with published results.

### Correlations with previous insect molecular data on desiccation and dehydration

There are no previous molecular studies on cryoprotective dehydration in any other organisms. The most closely related studies are those on insect desiccation stress and drought acclimation, but clearly the mechanisms between these survival tactics may differ and molecular analyses are very limited. Biochemical studies have so far shown two universal reactions to desiccation stress:

• Production of cryoprotectants e.g. trehalose and glycerol [12,13,18,74].

• Changes in membrane composition [13,18,86].

The two processes are linked, as removal of water profoundly affects the physical properties of membrane phospholipids and leads to destructive events such as fusion, phase transitions and increased permeability. The sugar-based cryoprotectants prevent damage from dehydration by inhibiting fusion between adjacent vesicles during desiccation and also by maintaining the lipids in a fluid state in the absence of water [87]. This protective process is substantiated in *M. arctica *by elevations in the expression levels of the desaturase gene and TPS (also reflected in biochemical studies [12]).

At the gene level, dehydration-induced expression of LEA proteins has been demonstrated in a chironomid midge (*Polypedilum vanderplanki*) [22] and differential regulation of HSPs in a flesh fly (*Sarcophaga crassipalpis*) [19] and a midge (*Belgica antarctica*) [17]. The latter two studies indicate the importance of not generalising results from a single species, as HSP23 and HSP70 are up-regulated in response to desiccation stress in the flesh fly pupae [19], whilst neither are up-regulated in the Antarctic midge [17]. Experiments on rehydration using the flesh fly pupae showed up-regulation of different HSPs compared with desiccation, via family members; HSP90 and the constitutive form of HSP70 (HSC70). *M. arctica *presents a different profile again with only the small HSP up-regulated in response to both desiccation and rehydration. However, the work on the Antarctic midge also measured osmolarity and showed no overall gain or loss of metabolites during desiccation, suggesting that osmolytes may have been redistributed from the haemolymph to intracellular compartments [17]. This is reflected in our studies by the altered expression of membrane-associated genes involved in solute and ion transport (c.f. aquaporins, a member of the cation transport ATPase family, caltexin (buffering intracellular Ca^2+^), a putative Na^+^/citrate co-transporter, SEC14 (catalysing the exchange of phospholipids between membranes) and a trehalose transporter.

Finally links can be made with the different approaches taken to examine constant and fluctuating cold temperatures in a parasitic wasp (*Aphidus colemani*) [88,89] and rapid cold hardening in the flesh fly (*S. crassipalpis*) [18]. In the first study, proteomics was used and of particular note was the up-regulation of arginine kinase as a means of energy production during recovery and TCP-1 (a sub-unit of chaperonin CCT) and a chitin binding protein during constant cold temperatures [88]. There was also a clear involvement of the protein-folding machinery and cytoskeletal rearrangement during both processes. Our studies show similar results with up-regulation of arginine kinase during the recovery treatment and continual involvement of cytoskeletal associated proteins during all treatments. The identification of enzymes involved in amino acid metabolism (cysteine dioxygenase and glutamate semialdehyde dehydrogenase) during the heat map analysis in *M. arctica *correlates with findings from metabolomics studies showing alterations in the free amino acid pool associated with stress treatment and recovery [18,89]. Glutamine regulation is of particular note as it is associated with both TPS expression (*M. arctica*) and rapid cold hardening (*S. crassipalpis*) [18]. This amino acid has been shown to not only contribute to osmolarity regulation, but also to potentially increase the responsiveness of heat shock proteins [90] and suppress apoptosis [91]. It has also previously been shown to accumulate in a number of other insect species in response to cold temperatures and diapause [92-95].

So, although molecular analyses in other species are limited and may not represent the same physiological mechanism, there are obvious similarities in response that can be drawn from a number of different types of analyses (including biochemical, proteomic and metabolomic). These, together with the data presented here are gradually increasing our knowledge on how insects successfully survive dehydration events.

## Conclusion

Microarray analysis has produced a greater understanding of the processes and genes underlying the process of cryoprotective dehydration. Namely production and mobilisation of trehalose, protection of cellular systems via small heat shock proteins and tissue/cellular remodelling during the desiccation process. Energy production, initiation of protein translation and cell division, plus potential tissue repair processes dominate genes identified during recovery. Q-PCR on selected candidate genes has also contributed to our understanding, with glutathione-S-transferase identified as the major antioxdidant enzyme protecting the cells during these stressful procedures and a number of protein kinase signalling molecules involved in recovery. Desaturase, a gene associated with changes in membrane composition also showed changes in expression levels with treatment. Heat map analysis of genes co-regulated with trehalose-6-phosphate synthase was particularly useful in identifying a number of candidate clones with sequence similarity to membrane proteins and signalling molecules, which will be targeted in future, more functionally based, analyses.

## Methods

### Sample collection and preparation

*M. arctica*, were collected under the bird cliffs at Stuphallet and Krykkefjellet on the Brøggerhalvøya, near Ny Ålesund, Spitsbergen, Svalbard, Norway (78°56'N, 11°53'E) and transported to the British Antarctic Survey (BAS), Cambridge, for analysis. Animals (mixture of both adult and juveniles) were cultured in ventilated plastic boxes containing moss, lichen and soil taken from field sites and fed on dried baker's yeast. Cultures were kept moist at +4°C.

### Microarray construction and hybridization

The 13,824 feature microarray was constructed by printing 6912 PCR-amplified cDNA clones in duplicate. These were derived from a previous EST study [26] and comprised 3840 clones from the Library D2 (fully dehydrated animals, cooled to -14°C) and 3072 clones from the Library D1 (dehydrating animals cooled to -2°C). The Stratagene SpotReport Alien Array Validation System (Stratagene, La Jolla, CA, USA) was included on the microarray. Construction and hybridization of the arrays were performed as previously described [96] with the exception of the amino-modified primers used for the initial cDNA amplification for array printing, which in this study were: (pAL32FOR: TTCTCGGGAAGCGCG and M13 forward: GTAAAACGACGGCCAG). Hybridizations were performed using control animals in combination with the groups listed below.

Five groups of animals were used for the hybridizations. Treatments were as follows:

• C = control = live animals from +5°C

• -2°C = cold dehydrated animals, cooled from +4°C to -2°C at a rate of 2°C/week and held at -2°C for 7 days. Animals were kept in sealed tubs on moist plaster of Paris. Ice chips were added to the tubs once the temperature reached below 0°C. Final water content of the animals was approximately 1.1 ± 0.2 g/g dry weight.

• -7°C = cold dehydrated animals, protocol as above, but cooled to -7°C. Final water content of the animals was approximately 0.57 ± 0.2 g/g dry weight.

• H18 = animals taken from -7°C, and left to recover for 18 hours at +5°C with moisture.

• 0.9 salt = salt dehydrated = animals were slowly dehydrated over a saturated solution of potassium nitrate (which gives a constant humidity of 96% RH at +5°C) to produce animals with a water content og 0.9 ± 0.12 g/g dry weight i.e. slightly less than the -2°C animals.

• 0.2 salt = salt dehydrated = animals were dehydrated as above to produce animals with a water content og 0.2 ± 0.07 g/g dry weight i.e. slightly less than the -7°C animals.

6 biological replicates and technical replicates in the form of dye swops were performed for each condition.

### Microarray data analysis

The microarray images were analysed using the GenePix 6.0 software (Molecular Devices). After gridding and segmentation, visual inspection was used to flag and exclude anomalous spots. The R [97] Limma package [98-100] was used for analysis. Background Subtraction was applied using the normexp function with an offset of 50 [101], within array normalisation through print-tip loess [102] and normalisation between arrays with Rquantile. Differentially expressed clones were selected at FDR adjusted p-value [103] of 0.01 and a B statistic (log-odds) of 4.5, giving a 99% probability of differential expression.

Annotation and gene ontology mapping of the clones was carried out as described in [27]. GO enrichment was determined by a proportion test, at a FDR adjusted p-value of 0.01, between the number of clones representing a GO term on the chip compared to the number of differentially expressed clones representing the same GO term in a given list. The array design is housed at ArrayExpress, accession number: A-MEXP-1540, and the experiments: E-MEXP-2105. All clones on the array are taken from a sub-set of the EST data set analysed in [27] (accession numbers: dbEST: 49109381–49125759, Genbank: EW744731–EW761109). From a complete cross correlation matrix, 53 clones having 98% correlation or more with the 3 trehalose clones (sb_006_04P17, sb_009_07N11, sb_006_06G01) were pulled out and the log fold changes of the treatments were used to construct a heat map using hierarchical clustering with Euclidean distance measure and Average linkage (UPGMA) [104]. A similar approach was used to cluster all the post-analysis data to construct the dendrogram in Figure 3.

### Isocitrate lyase PCR

A primer pair was chosen to span approximately 800 bp of the EST contig. Forward primer: TGAACGTCGCTATACTGCTG; Reverse primer: ATATGGTGCGTAAGCCAAAC. PCR conditions for both genomic DNA and cDNA (produced from -2°C cold dehydrated animals): 95°C 10 mins, 35 cycles of 95°C 30 secs, 62°C 30 secs, 72°C 3 mins, final extension of 72°C 10 mins using standard PCR mix (BioTaq from Bioline) with 15 mM MgCl_2_.

### Q-PCR

To validate the microarray results, 21 clones were chosen for Q-PCR analysis (Table 3). The RSq and efficiency values were calculated for each primer set (Table 3). All genes were amplified using specific primers, Brilliant SYBR^® ^Green QPCR Master Mix (Stratagene) and an MX3000P Q-PCR machine (Stratagene). PCR conditions were as follows: 95°C 10 minutes, 40 cycles of 95°C 30 seconds, 60°C 1 minute and 72°C for 45 seconds with a final dissociation curve step as per manufacturer's recommendations. The plate set-up for each Q-PCR experiment consisted of the 5 animal treatments (including controls) amplified in triplicate (technical replicates) with the clone sb_009_01G07 used as a control sequence. This clone had been previously identified in the preliminary microarray analyses as invariant between all treatments and showed no sequence similarity to any characterized gene in the public databases. Primers were validated and the results analysed as described in [82] using the methods of [105,106], which incorporates the efficiency of the primers as a factor in the equation. The Q-PCR analyses were all carried out on freshly isolated RNA and cDNA, hence representing different biological replicates compared to the RNAs used for the microarrays.

**Table 3:** Q-PCR primers with RSq and efficiency values

**Gene Name**	**Clone ID**	*Primer Sequence*	**RSq**	**% Efficiency**
Housekeeping sequence				

Unknown	sb_009_01G07	F: CTCGGACTCAGCCTGTCTA R: ATAAATCGGACTTCCATTTCCA	0.982	95

				

**Aquaporins**				

AQP-A	sb_006_02P03	F: ATGTTGTAGGAGGAAGCGTCA R: ACAAACCGTCGTCTCGGTAG	0.991	115

AQP-B	sb_006_05H07	F: ATTGGTGCTTGGTTGTTGAA R: AATCCTGCTCCAGTGAATCC	0.996	98

AQP-C	sb_006_08O08	F: TACCGTCGCTTCCATGATTA R: CCCTTCACGTAGCTCCAGTT	0.995	105

				

**Heat shock proteins**				

sHSP	sb_009_11D21	F: ACTCCACGCCAGCATTTCTTC R: TCCAATTCTGTCCGCATTATTCC	0.991	111

HSP70	sb_009_03K12	F: CAAGGAGGTGGACAACAACAATC R: CACAATCATTCAGCAGCAATAACAC	0.994	92

				

**Antioxidants**				

Superoxide dismutase	sb_009_01L22	F: ACGAGAAGGTTGACGATTTGGG R: ATTCCGCAGCCTAAACGAGAC	0.998	86

Catalase	sb_006_03D21	F: CCCGAGATGGATTTATGAGTCC R: CTAAGTACACAGAAGCCACACC	0.991	183

Glutathione-S-transferase	sb_006_04F01	F: TGCTCCAAGTCGTGCCGTTC R: GATGGCTCGTGACTCGCTTAG	0.988	153

				

**Various**				

Ferritin	sb_006_04H15	F: GGAGGTCGTGTCGTTCTTCAG R: CAGTTGTGGGTCACCATTTCG	0.991	94

Desaturase	sb_006_09F19	F: GCTCCTGACCCGTAAACATCC R: CCATGCTGCCAATAAACTTTCACC	0.996	105
				

				
**Trehalose GO annotations**				

Trehalose 6 phosphate synthase (1)	sb_006_04P17	F: TGAATTGGACGATTACGCTGAAG R: AGACTGCCCATTGCTTTGAGG	1.00	86

Trehalose 6 phosphate synthase (2)	sb_006_06G01	F: GGATGGAATTACTGGAGCTTGG R: GTGCTTGATGAGCTGTGAAACC	0.985	110

LATS tumour supressor	sb_006_04E02	F: CGGGACGGACATATCAAACT R: TGAGCAAGGCATCGTACATT	0.995	97

Serine/threonine protein kinase 38	sb_006_04N08	F: GACTGGTGGAGTCTGGGAGT R: CAGTCCACGCTTTTGAAGAA	0.992	122

Protein kinase A cAMP dependant catalytic sub-unit	sb_006_07A12	F: TCAAAGGTCGAACCTGGACT R: AACCGTACTTTCCCTGCAAC	0.990	100

Trehalase precursor	sb_006_01B14	F: CCGTAGATGGACTTCCTGGT R: TGCCTGTCAGAACACACAAA	0.985	117

P70 ribosomal protein S6 kinase	sb_006_01P13	F: GGGCGAGATGCTAATGAAAT R: CCTTCGTGTGAGCAGTGTCT	0.991	115

Putative protein kinase DC2	sb_006_05C18	F: ATGGAGAATGGCACTGAGGAC R: CTTAGGCGTCTTTGGTAAACATCC	0.995	99

Similar: serine/threonine protein kinase 6 (Aurora family kinase 1)	sb_006_07A19	F: ACTTTGACATTGGGCGTCTC R: CACCAGGGGCATATTCAAGA	0.934	180

Protein kinase C	sb_008_03M09	F: ACTCCACGATGATGTGTTGTATCC R: TCCAGAACCACTTCCTTGATTGC	0.995	80

cAMP dependant protein kinase C1	sb_009_02D08	F: CAACGTCATCTACCGTGACC R: AAATACTCCGGTGTCCCACA	0.988	102

## Authors' contributions

MC was a BAS Co-PI on the external funding, drafted the manuscript and lead the analyses. MAST performed the data analyses. JP amplified the clones for the microarray and performed many of the hybridizations and the initial quality control of experiments. GB supervised the production of the clones for the microarray, produced the final microarrays, performed some of the hybridizations and supervised the quality control. ŽP performed the Q-PCR analyses. G-G-L assisted with the biological interpretation of the analyses. MRW was a BAS Co-PI on the external funding, performed the physiological experiments to produce the animals under different conditions, conducted biochemical analyses and contributed to drafting the manuscript. MSC, MAST, JP, G-GL and MRW were all involved in fieldwork and animal collection over the period of the project. All authors read and approved the final manuscript.

## Supplementary Material

Additional file 1 **The "Top 20" sequenced up-regulated clones in the -2°C cold dehydration experiment, with putative functionality assigned via BLAST sequence similarity searching.** All matches are in excess of 1.0 e^-10 ^unless stated in the discussion. **Definitions: LogFold** = Estimate of the log2-fold change corresponding to the effect or contrast; **AveExpr** = Average log2 expression for the probe over all arrays and channels; adj p val = adjusted as described in methods; **B** = log odds that the gene is differentially expressed. BLAST sequence similarity data. Click here for file

Additional file 2 **The "Top 20" sequenced up-regulated clones in the -7°C cold dehydration experiment, with putative functionality assigned via BLAST sequence similarity searching**. All matches are in excess of 1.0 e^-10 ^unless stated in the discussion. Detail of columns: as for Additional file 1. BLAST sequence similarity data. Click here for file

Additional file 3 **The "Top 20" sequenced up-regulated clones in the 0.9 salt dehydrated experiment, with putative functionality assigned via BLAST sequence similarity searching**. All matches are in excess of 1.0 e^-10 ^unless stated in the discussion. Detail of columns: as for Additional file 1. BLAST sequence similarity data. Click here for file

Additional file 4 **The "Top 20" sequenced up-regulated clones in the 0.2 salt dehydrated experiment, with putative functionality assigned via BLAST sequence similarity searching**. All matches are in excess of 1.0 e^-10 ^unless stated in the discussion. Detail of columns: as for Additional file 1. BLAST sequence similarity data. Click here for file

Additional file 5 **The "Top 20" sequenced up-regulated clones in the 18 hour recovery experiment, with putative functionality assigned via BLAST sequence similarity searching**. All matches are in excess of 1.0 e^-10 ^unless stated in the discussion. Detail of columns: as for Additional file 1. BLAST sequence similarity data. Click here for file

Additional file 6 **Clones co-regulated with TPS across all four dehydration treatments, with putative functionality assigned via BLAST sequence similarity searching**. All matches are in excess of 1.0 e^-10 ^unless stated in the discussion. BLAST sequence similarity data. Click here for file
