# Supplementary material for: Surviving the cold: molecular analyses of insect cryoprotective dehydration in the Arctic springtail Megaphorura arctica (Tullberg)
Source: BMC Genomics. 2009 Jul 21;10:328. doi: 10.1186/1471-2164-10-328 (PMC2726227; doi:10.1186/1471-2164-10-328)
Supplement: Additional file 1 — The "Top 20" sequenced up-regulated clones in the -2°C cold dehydration experiment, with putative functionality assigned via BLAST sequence similarity searching. All matches are in excess of 1.0 e-10 unless stated in the discussion. Definitions: LogFold = Estimate of the log2-fold change corresponding to the effect or contrast; AveExpr = Average log2 expression for the probe over all arrays and channels; adj p val = adjusted as described in methods; B = log odds that the gene is differentially expressed. BLAST sequence similarity data. [file 1471-2164-10-328-S1.doc]

**Additional file 1:** The “Top 20” sequenced up-regulated clones in the -2°C cold dehydration experiment, with putative functionality assigned via BLAST sequence similarity searching. All matches are in excess of 1.0 e^-10^ unless stated in the discussion. **Definitions:** **LogFold** = Estimate of the log2-fold change corresponding to the effect or contrast; **AveExpr** = Average log2 expression for the probe over all arrays and channels; **adj p val** = adjusted as described in methods; **B** = log odds that the gene is differentially expressed.

| Clone | **LogFold** | **AveExpr** | **adj.p.val** | **B** | Accession number | Gene identification | Putative function based on BLAST homology |
| --- | --- | --- | --- | --- | --- | --- | --- |
| sb_006_05O02 | 3.09 | 11.13 | 2.35E-033 | 74.15 |  | No significant match |  |
| sb_009_11N15 | 2.95 | 10.31 | 1.51E-031 | 69.38 |  | No significant match |  |
| sb_006_08N11 | 2.55 | 10.58 | 4.22E-030 | 65.69 |  | No significant match |  |
| sb_006_04P15 | 2.98 | 11.37 | 7.67E-029 | 62.31 | Q9VDR1 | Mediator of RNA polymerase II transcription sub-unit | Transcriptional regulation |
| sb_006_06A18 | 1.98 | 11.41 | 1.54E-028 | 61.44 |  | No significant match |  |
| sb_006_06P19 | 3.00 | 10.52 | 1.69E-028 | 61.23 |  | No significant match |  |
| sb_006_06G01 | 2.71 | 10.67 | 2.01E-028 | 60.89 | A3EY17 | Trehalose-6-phosphate synthase | Trehalose synthesis |
| sb_009_02N23 | 2.75 | 10.95 | 2.59E-028 | 60.51 |  | No significant match |  |
| sb_009_12A22 | 2.68 | 11.54 | 8.50E-028 | 59.23 |  | No significant match |  |
| sb_006_08A03 | 2.14 | 11.48 | 1.54E-027 | 58.57 |  | No significant match |  |
| sb_006_09L15 | 2.95 | 11.39 | 2.82E-027 | 57.79 | Q179R1 | Putative uncharacterised protein in *Aedes aegypti* | Unknown |
| sb_006_07F11 | 2.20 | 12.58 | 2.82E-027 | 57.79 | Q5DLW4 | Putative dehydrogenase |  |
| sb_006_05O20 | 2.36 | 10.69 | 3.38E-027 | 57.57 |  | No significant match |  |
| sb_006_08I06 | 2.85 | 10.53 | 4.11E-027 | 57.26 | Q179R1 | Putative uncharacterised protein in *Aedes aegypti* | Unknown |
| sb_009_06B07 | 2.52 | 9.66 | 1.30E-026 | 56.01 |  | No significant match |  |
| sb_009_12P06 | 2.52 | 10.27 | 1.99E-026 | 55.52 | Q16JG6 | Small heat shock protein | Stress protein |
| sb_006_01F04 | 2.68 | 10.35 | 2.68E-026 | 55.10 | Q16KG8 | Putative uncharacterised protein in *Aedes aegypti* | Unknown |
| sb_006_01E17 | 2.18 | 10.73 | 1.33E-025 | 53.40 |  | No significant match |  |
| sb_006_08J22 | 3.13 | 10.85 | 1.67E-025 | 53.12 |  | No significant match |  |
| sb_006_10I11 | 2.54 | 13.54 | 2.32E-025 | 52.75 | Q16JG6 | Small heat shock protein | Stress protein |
| No sequence | sb_006_06H03 | | | | | | |
| Bacterial | sb_006_05N21, sb_006_08M03, sb_009_07J01 | | | | | | |
