# Supplementary material for: Surviving the cold: molecular analyses of insect cryoprotective dehydration in the Arctic springtail Megaphorura arctica (Tullberg)
Source: BMC Genomics. 2009 Jul 21;10:328. doi: 10.1186/1471-2164-10-328 (PMC2726227; doi:10.1186/1471-2164-10-328)
Supplement: Additional file 2 — The "Top 20" sequenced up-regulated clones in the -7°C cold dehydration experiment, with putative functionality assigned via BLAST sequence similarity searching. All matches are in excess of 1.0 e-10 unless stated in the discussion. Detail of columns: as for Additional file 1. BLAST sequence similarity data. [file 1471-2164-10-328-S2.doc]

**Additional file 2:** The “Top 20” sequenced up-regulated clones in the -7°C cold dehydration experiment, with putative functionality assigned via BLAST sequence similarity searching. All matches are in excess of 1.0 e^-10^ unless stated in the discussion. Detail of columns: as for Additional file 1.

| **Clone** | **LogFold** | **AveExpr** | **adj.p.val** | **B** | **Accession** **number** | **Gene identification** | **Putative function based on BLAST homology** |
| --- | --- | --- | --- | --- | --- | --- | --- |
| sb_006_06P19 | 3.52 | 10.52 | 3.94E-032 | 70.06 |  | No significant match |  |
| sb_009_07N09 | 3.22 | 10.38 | 4.75E-032 | 69.66 |  | No significant match |  |
| sb_009_06B07 | 2.74 | 9.665 | 5.56E-030 | 64.52 |  | No significant match |  |
| sb_006_05O20 | 2.67 | 10.69 | 6.13E-030 | 64.28 |  | No significant match |  |
| sb_009_02I23 | 2.53 | 10.95 | 1.18E-028 | 61.03 |  | No significant match |  |
| sb_009_12E12 | 3.42 | 11.14 | 3.06E-027 | 57.02 | Q29KQ1 | Ubiquitin carrier protein | Proteolysis |
| sb_006_02N12 | 2.57 | 12.16 | 5.67E-027 | 56.36 |  | No significant match |  |
| sb_006_08J20 | 2.72 | 10.35 | 4.46E-026 | 54.23 | A9ZSY1 | Trehalose transporter | Sugar transport |
| sb_006_08A03 | 1.95 | 11.48 | 6.08E-026 | 53.79 |  | No significant match |  |
| sb_006_09P19 | 2.52 | 11.31 | 1.26E-025 | 52.92 | Q07152 | Inosine 5’ monophosphate dehydrogenase | Regulation of cell growth |
| sb_006_08N10 | 2.35 | 10.64 | 1.46E-025 | 52.67 | P48603 | F-capping protein | Cytoskeleton |
| sb_006_05G17 | 2.05 | 11.16 | 1.46E-025 | 52.64 |  | No significant match |  |
| sb_006_08J22 | 3.07 | 10.85 | 2.25E-025 | 52.13 |  | No significant match |  |
| sb_006_04P15 | 2.44 | 11.37 | 5.59E-025 | 51.12 |  | No significant match |  |
| sb_006_06G05 | 2.81 | 10.94 | 6.84E-025 | 50.85 | Q9VDR1 | Mediator of RNA polymerase II transcription sub-unit | Transcriptional regulation |
| sb_006_10K06 | 2.50 | 12.01 | 1.58E-024 | 49.87 | Q05BV0 | AKT2 | Cytoskeleton |
| sb_006_10H15 | 2.10 | 10.72 | 5.35E-024 | 48.62 | Q7PIQ3 | TER94 | Proteolysis |
| sb_006_10F03 | 2.21 | 11.05 | 5.35E-024 | 48.55 |  | No significant match |  |
| sb_009_12I04 | 1.84 | 11.34 | 2.57E-023 | 46.86 |  | No significant match |  |
| sb_006_04L01 | 2.39 | 11.52 | 2.64E-023 | 46.80 | A5HSI6 | Juvenile hormone esterase | Regulator of juvenile hormone |
| No sequence | sb_006_06K07 | | | | | | |
