# Supplementary material for: Surviving the cold: molecular analyses of insect cryoprotective dehydration in the Arctic springtail Megaphorura arctica (Tullberg)
Source: BMC Genomics. 2009 Jul 21;10:328. doi: 10.1186/1471-2164-10-328 (PMC2726227; doi:10.1186/1471-2164-10-328)
Supplement: Additional file 3 — The "Top 20" sequenced up-regulated clones in the 0.9 salt dehydrated experiment, with putative functionality assigned via BLAST sequence similarity searching. All matches are in excess of 1.0 e-10 unless stated in the discussion. Detail of columns: as for Additional file 1. BLAST sequence similarity data. [file 1471-2164-10-328-S3.doc]

**Additional file 3:** The “Top 20” sequenced up-regulated clones in the 0.9 salt dehydrated experiment, with putative functionality assigned via BLAST sequence similarity searching. All matches are in excess of 1.0 e^-10^ unless stated in the discussion. Detail of columns: as for Table 2.

| **Clone** | **LogFold** | **AveExpr** | **adj.p.val** | **B** | **Accession** **number** | **Gene identification** | **Putative function based on BLAST homology** |
| --- | --- | --- | --- | --- | --- | --- | --- |
| sb_006_09K03 | 2.266941 | 10.150392 | 2.80E-023 | 50.06 | Q23O23 | Unc-51 | Protein kinase signalling |
| sb_009_02E21 | 1.718358 | 11.961502 | 5.90E-023 | 48.82 | O42200 | Tec-family kinase | Protein kinase signalling |
| sb_006_08B24 | 1.745269 | 10.829149 | 5.02E-021 | 43.97 | A7SPB7 | Tolloid protein | Protease |
| sb_009_07O06 | 1.705908 | 11.789018 | 6.91E-021 | 43.53 | Q3MQO7 | Autophagy protein | Proteolysis |
| sb_006_08F04 | 1.647749 | 11.149380 | 7.27E-021 | 43.37 | P48428 | TCP-1 | Chaperone |
| sb_006_05O02 | 1.910645 | 11.131971 | 1.34E-020 | 42.59 |  | No significant match |  |
| sb_006_07L15 | 3.613726 | 11.182903 | 2.84E-020 | 41.65 | BOWK59 | Transmembrane protein | Uncharacterised transmembrane protein |
| sb_006_09G11 | 1.575578 | 11.159224 | 4.04E-020 | 41.16 |  | No significant match |  |
| sb_006_03H12 | 2.221515 | 11.277013 | 4.12E-020 | 41.07 |  | No significant match |  |
| sb_006_06G01 | 1.835329 | 10.665258 | 1.14E-019 | 39.87 | A3EY17 | Trehalose-6-phosphate synthase | Trehalose synthesis |
| sb_006_09D02 | 1.567005 | 12.287682 | 1.49E-019 | 39.47 |  | No significant match |  |
| sb_006_01E17 | 1.674874 | 10.726173 | 1.73E-019 | 39.27 |  | No significant match |  |
| sb_006_09L17 | 2.035860 | 10.147138 | 2.05E-019 | 39.10 | B1A650 | Cytochrome p450 | Detoxification |
| sb_009_04F07 | 1.371833 | 10.583514 | 2.70E-019 | 38.64 | Q0N2S1 | Nucleolysin TIAR protein | Apoptosis |
| sb_009_04J23 | 1.736980 | 11.842124 | 8.85E-019 | 37.30 |  | No significant match |  |
| sb_006_04P15 | 1.869595 | 11.373198 | 9.07E-019 | 37.25 | Q9VDR1 | Mediator of RNA polymerase II transcription sub-unit | Transcriptional regulation |
| sb_006_04B18 | 1.538111 | 11.095499 | 1.16E-018 | 36.97 |  | No significant match |  |
| sb_006_01H11 | 1.195554 | 12.333616 | 1.72E-018 | 36.49 | O77402 | Elongation factor 1 α | Transcription |
| sb_006_08B11 | 1.488478 | 11.580385 | 2.31E-018 | 36.13 | Q59GS3 | Proteasome 26s ATPase | Proteolysis |
| sb_006_08M21 | 1.748162 | 11.941702 | 3.37E-018 | 35.71 |  | No significant match |  |
