# Supplementary material for: Surviving the cold: molecular analyses of insect cryoprotective dehydration in the Arctic springtail Megaphorura arctica (Tullberg)
Source: BMC Genomics. 2009 Jul 21;10:328. doi: 10.1186/1471-2164-10-328 (PMC2726227; doi:10.1186/1471-2164-10-328)
Supplement: Additional file 4 — The "Top 20" sequenced up-regulated clones in the 0.2 salt dehydrated experiment, with putative functionality assigned via BLAST sequence similarity searching. All matches are in excess of 1.0 e-10 unless stated in the discussion. Detail of columns: as for Additional file 1. BLAST sequence similarity data. [file 1471-2164-10-328-S4.doc]

**Additional file 4:** The “Top 20” sequenced up-regulated clones in the 0.2 salt dehydrated experiment, with putative functionality assigned via BLAST sequence similarity searching. All matches are in excess of 1.0 e^-10^ unless stated in the discussion. Detail of columns: as for Additional file 1.

| **Clone** | **LogFold** | **AveExpr** | **adj.p.val** | **B** | **Accession** **number** | **Gene identification** | **Putative function based on BLAST homology** |
| --- | --- | --- | --- | --- | --- | --- | --- |
| sb_009_07N09 | 2.87 | 10.38 | 1.89E-029 | 63.41 |  | No significant match |  |
| sb_006_05O20 | 2.26 | 10.69 | 1.73E-026 | 55.20 |  | No significant match |  |
| sb_006_01E17 | 2.22 | 10.73 | 3.38E-026 | 54.38 |  | No significant match |  |
| sb_006_02O13 | 2.25 | 10.84 | 9.02E-026 | 53.21 |  | No significant match |  |
| sb_006_08A17 | 1.81 | 12.04 | 2.88E-025 | 51.90 |  | No significant match |  |
| sb_006_09C13 | 1.58 | 11.59 | 4.09E-025 | 51.50 |  | No significant match |  |
| sb_006_06G01 | 2.21 | 10.67 | 2.08E-024 | 49.73 | A3EY17 | Trehalose-6-phosphate synthase | Trehalose synthesis |
| sb_006_02N12 | 2.25 | 12.16 | 3.88E-024 | 48.88 |  | No significant match |  |
| sb_006_07D01 | 1.50 | 11.48 | 1.74E-023 | 47.26 |  | No significant match |  |
| sb_009_02I23 | 1.89 | 10.95 | 1.11E-022 | 45.20 |  | No significant match |  |
| sb_009_06B07 | 1.79 | 9.665 | 1.86E-022 | 44.81 |  | No significant match |  |
| sb_006_08J22 | 2.64 | 10.85 | 3.11E-022 | 44.07 |  | No significant match |  |
| sb_006_04P15 | 2.12 | 11.37 | 4.37E-022 | 43.71 | Q9VDR1 | Mediator of RNA polymerase II transcription sub-unit | Transcriptional regulation |
| sb_006_06G05 | 2.44 | 10.94 | 7.12E-022 | 43.17 |  | No significant match |  |
| sb_006_10K06 | 2.20 | 12.01 | 7.61E-022 | 43.09 | A8MX96 | AKT2 | Cytoskeleton |
| sb_006_10H15 | 1.87 | 10.72 | 9.75E-022 | 42.90 | Q7PIQ3 | TER94 | Proteolysis |
| sb_009_04J09 | 1.88 | 10.16 | 2.03E-021 | 42.05 |  | Putative transporter | Membrane transport |
| sb_009_11N15 | 1.71 | 10.31 | 5.71E-021 | 40.90 |  | No significant match |  |
| sb_009_07C13 | 1.58 | 11.45 | 1.06E-020 | 40.16 |  | No significant match |  |
| sb_009_07B12 | 2.08 | 8.485 | 1.51E-020 | 40.01 |  | No significant match |  |
| No sequence | sb_009_11M13, sb_006_09G11, sb_006_09O04, sb_006_07J22, sb_006_09N12, sb_006_03B19, sb_006_08J20, sb_006_01K02, sb_009_11G22, sb_006_F03, sb_009_12I04 | | | | | | |
| Bacterial | sb_006_05N21, sb_009_07J01, sb_006_08M03, sb_006_03I18, sb_009_12H05 | | | | | | |
