# Supplementary material for: Surviving the cold: molecular analyses of insect cryoprotective dehydration in the Arctic springtail Megaphorura arctica (Tullberg)
Source: BMC Genomics. 2009 Jul 21;10:328. doi: 10.1186/1471-2164-10-328 (PMC2726227; doi:10.1186/1471-2164-10-328)
Supplement: Additional file 5 — The "Top 20" sequenced up-regulated clones in the 18 hour recovery experiment, with putative functionality assigned via BLAST sequence similarity searching. All matches are in excess of 1.0 e-10 unless stated in the discussion. Detail of columns: as for Additional file 1. BLAST sequence similarity data. [file 1471-2164-10-328-S5.doc]

**Additional file 5:** The “Top 20” sequenced up-regulated clones in the 18 hour recovery experiment, with putative functionality assigned via BLAST sequence similarity searching. All matches are in excess of 1.0 e^-10^ unless stated in the discussion. Detail of columns: as for Additional file 1.

| **Clone** | **LogFold** | **AveExpr** | **adj.p.val** | **B** | **Accession** **number** | **Gene identification** | **Putative function based on BLAST homology** |
| --- | --- | --- | --- | --- | --- | --- | --- |
| sb_006_05M08 | 2.2 | 11.88 | 2.35E-028 | 59.65 |  | No significant match |  |
| sb_006_09D01 | 3.13 | 11.80 | 4.22E-028 | 58.91 |  | No significant match |  |
| sb_006_03O18 | 2.02 | 13.03 | 5.17E-028 | 58.67 | P36194 | HMGB1 | Regulation of transcription |
| sb_006_02J05 | 2.28 | 12.29 | 3.84E-027 | 56.21 |  | No significant match |  |
| sb_006_04B23 | 2.12 | 15.00 | 8.00E-027 | 55.36 | P36194 | HMGB1 | Regulation of transcription |
| sb_006_09L16 | 2.41 | 11.75 | 1.28E-026 | 54.82 |  | No significant match |  |
| sb_006_10D11 | 2.29 | 11.50 | 1.74E-026 | 54.44 | Q5XUU6 | Take-out carrier protein | Protein with unknown function, juvenile hormone binding motif |
| sb_006_07F23 | 2.44 | 13.61 | 2.63E-026 | 53.95 | B4GEC2 | Metalloproteinase | Proteolysis |
| sb_006_04F21 | 2.16 | 13.30 | 2.85E-026 | 53.85 |  | No significant match |  |
| sb_006_07F19 | 2.38 | 11.75 | 1.01E-025 | 52.5 | A8CWD0 | Chitin-binding protein | Cytoskeletal |
| sb_006_07B15 | 2.73 | 11.96 | 9.67E-026 | 52.48 | Q10475 | Eukaryotic translation initiation factor 4 γ | mRNA translation |
| sb_006_03P20 | 2.00 | 13.10 | 1.51E-025 | 52.03 | Q3SZH7 | Leukotriene A-4 hydrolase | Amino peptidase and epoxide hydrolase activity |
| sb_009_04F14 | 2.47 | 11.22 | 1.44E-025 | 52.02 |  | No significant match |  |
| sb_006_07O14 | 2.27 | 11.70 | 3.26E-025 | 51.14 | B1A4C9 | Arginine kinase | Energy shuttle |
| sb_006_10C08 | 1.82 | 12.11 | 9.50E-025 | 50.01 | Q5D9B5 | Putative uncharacterised protein in *Schistosoma japonicum* |  |
| sb_006_10O17 | 1.92 | 12.34 | 1.90E-024 | 49.27 | Q6X6Z7 | Tektin 3 | Cytoskeleton |
| sb_006_04G22 | 4.00 | 15.73 | 2.37E-024 | 49.13 |  | No significant match |  |
| sb_006_10G01 | 2.27 | 11.81 | 4.44E-024 | 48.35 | P91944 | Elongation factor 1 α-like factor | Transcription |
| sb_006_09J07 | 2.06 | 11.99 | 1.01E-023 | 47.44 |  | No significant match |  |
| sb_006_09H05 | 3.02 | 11.93 | 1.37E-023 | 47.11 | A8CWD0 | Chitin-binding protein | Cytoskeletal |
| No sequence | sb_006_02E15, sb_006_09N06, sb_009_07G15, sb_006_09D22, sb_006_05F12 | | | | | | |
