# Supplementary material for: Surviving the cold: molecular analyses of insect cryoprotective dehydration in the Arctic springtail Megaphorura arctica (Tullberg)
Source: BMC Genomics. 2009 Jul 21;10:328. doi: 10.1186/1471-2164-10-328 (PMC2726227; doi:10.1186/1471-2164-10-328)
Supplement: Additional file 6 — Clones co-regulated with TPS across all four dehydration treatments, with putative functionality assigned via BLAST sequence similarity searching. All matches are in excess of 1.0 e-10 unless stated in the discussion. BLAST sequence similarity data. [file 1471-2164-10-328-S6.doc]

**Additional file 6:** Clones co-regulated with TPS across all four dehydration treatments, with putative functionality assigned via BLAST sequence similarity searching. All matches are in excess of 1.0 e^-10^ unless stated in the discussion.

| **Clone** | **Accession**  **number** | **Putative gene/domain identification** | **Function** |
| --- | --- | --- | --- |
| sb_009_01K08 | A8C9W7 | ADP-ribosyl factor-like protein | Cell signalling |
| sb_006_04J14 | BOWJH4 | Juvenile hormone inducible protein | Protein of unknown function induced by juvenile hormone |
| sb_009_04N24 | Q9OWC3 | CathepsinB | Proteolysis |
| sb_006_06J06 | Q6WNX4 | Ferritin | Oxidoreductase |
| sb_009_12G13 | Q5T724 | DBH-like monooxygenase protein | Intracellular communication |
| sb_006_08L20 | Q5T724 | DBH-like monooxygenase protein | Intracellular communication |
| sb_006_06G01 | A3EY17 | Trehalose-6-phosphate synthase | Trehalose production |
| sb_006_01K07 | BOX420 | SEC14 | Membrane transport |
| sb_006_01F05 | Q9DFP6 | Cysteine dioxygenase | Amino acid metabolism |
| sb_009_07O14 | Q01EU3 | Centaurin α | Cell signalling |
| sb_009_06O13 | A2SXS9 | Putative zinc finger | Transcriptional regulation |
| sb_009_04F07 | Q0N2S1 | Nucleolysin TIAR protein | Apoptosis |
| sb_009_07N11 | A8D372 | Trehalose-6-phosphate synthase | Trehalose production |
| sb_006_04P17 | A8D372 | Trehalose-6-phosphate synthase | Trehalose production |
| sb_006_04L08 | Q16XE5 | Cation-transporting ATPase | Ion transport |
| sb_009_07D08 | A8CWD0 | Chitin-binding protein | Cytoskeletal |
| sb_009_05K08 | B4E181 | Solute carrier family 13 member | Membrane transport |
| sb_006_10P09 | A8CWD0 | Chitin-binding protein | Cytoskeletal |
| sb_006_03A01 | Q7PY19 | Uncharacterised protein in various insect species | Unknown |
| sb_006_07H22 | A8CWD0 | Chitin-binding protein | Cytoskeletal |
| sb_006_08F21 | A8CWD0 | Chitin-binding protein | Cytoskeletal |
| sb_006_03F06 | Q7YU97 | V-type proton ATPase 16Kda proteolipid subunit | Ion regulation |
| sb_006_08J23 | BOX119 | Glutamate semialdehyde dehydrogenase | Amino acid biosynthesis |
| sb_009_04F08 | B3UYC2 | Winged helix/forkhead transcription factor | Transcriptional regulation |
| sb_006_01B14 | Q9BLC8 | Trehalase precursor | Trehalose breakdown |
| sb_006_03D06 | O16158 | Calexcitin 2 | Calcium activated signalling molecule |
| **No database match** | sb_006_04E12, sb_006_01M05, sb_006_07D22, sb_009_04P07, sb_009_07M16, sb_006_05H11, sb_006_08J22, sb_006_10I15, sb_006_07J04, sb_006_08M19, sb_006_05E24, sb_006_05E16, sb_009_06H01, sb_006_06C16, sb_006_08C16, sb_006_03L22, sb_006_08O12, sb_006_06O15, sb_009_02L10, sb_009_03P19, sb_006_01E17, sb_009_04H04, sb_006_03C19, sb_006_10N01, sb_006_04M13 | | |
| **Unsequenced** | sb_009_04B09, sb_009_06N04, sb_009_03B09, sb_006_06M09, sb_006_03B19 | | |
